# Reported adverse events related to use of hepatitis C virus direct-acting antivirals with opioids: 2017–2021

**DOI:** 10.1186/s12954-023-00874-y

**Published:** 2023-10-01

**Authors:** Anthony Martinez, Tipu Khan, Douglas E. Dylla, John Marcinak, Michelle Collins, Brad Saget, Brian Conway

**Affiliations:** 1 Department of Medicine, Jacobs School of Medicine, University at Buffalo, State University of New York, 955 Main Street, Buffalo, NY 14203 USA; 2 Ventura County Medical Center, Ventura, CA USA; 3 US Medical Affairs – Virology, AbbVie Inc., North Chicago, IL USA; 4 Infectious Diseases Therapeutic Area, AbbVie Inc., North Chicago, IL USA; 5 Global Medical Affairs, AbbVie Inc., North Chicago, IL USA; 6 Global Medical Affairs – Virology/Hepatology, AbbVie Inc., North Chicago, IL USA; 7 Vancouver Infectious Diseases Centre, Vancouver, Canada; 8 Simon Fraser University, Burnaby, Canada

**Keywords:** Addiction, Clinical populations, Hepatitis, Safety, Substance abuse

## Abstract

**Introduction:**

Due to concerns over potential interactions between some hepatitis C direct-acting antivirals (DAAs) and opioids, we describe adverse event (AE) reports of concomitant use of opioids and DAAs.

**Methods:**

AEs reported (July 28, 2017–December 31, 2021) with the administration of the DAAs glecaprevir/pibrentasvir, sofosbuvir/velpatasvir, ledipasvir/sofosbuvir, sofosbuvir/velpatasvir/voxilaprevir, and elbasvir/grazoprevir as suspect products were downloaded from the US Food and Drug Administration AE Reporting System Public Dashboard. The number of AE reports containing opioids (fentanyl, hydrocodone, oxycodone) as co-suspect products/concomitant products were counted and summarized by severity, reporting country and whether an outcome of death was reported. Overdose AEs were counted irrespective of opioid use, and changes over time were assessed.

**Results:**

In total, 40 AEs were reported for DAAs and concomitant fentanyl use, 25 (62.5%) were in the USA, 35 (87.5%) were considered serious, and 14 (35.0%) resulted in death; and 626 were reported with concomitant oxycodone/hydrocodone use, 596 (95.2%) were in the USA, 296 (47.3%) were considered serious, and 28 (4.5%) resulted in death. There were 196 overdose AEs (32 [16%] deaths) declining from 2018 (*N* = 56) to 2021 (*N* = 29).

**Conclusions:**

Treating people with hepatitis C virus (HCV) infection who use drugs is key to achieving HCV elimination. Low numbers of DAA AE reports with opioids may provide reassurance to prioritize HCV treatment in this population. These data contribute to evidence supporting the continued scale-up of DAA treatment among people who use drugs to achieve HCV elimination goals.

**Supplementary Information:**

The online version contains supplementary material available at 10.1186/s12954-023-00874-y.

## Introduction

With the emergence of highly effective and well-tolerated pangenotypic direct-acting antivirals (DAAs) for hepatitis C virus (HCV) infection such as glecaprevir/pibrentasvir (G/P) and sofosbuvir/velpatasvir (SOF/VEL), achieving the World Health Organization’s (WHO) goal of global HCV elimination is now a possibility. However, rates of transmission of HCV infection remain high in populations such as people who inject drugs (PWID). In this population, it is estimated that 39.4% are infected with HCV, and 1 in 8 people are coinfected with HIV. Additionally, PWID are affected by mental health disorders such as depression with approximately 28.7% having a depression diagnosis [1–3]. In recent years, fentanyl has increasingly invaded the illicit drug supply market resulting in marked increases in overdoses, particularly in North America [4]. In the USA, an estimated 100,000 overdose deaths were reported in 2021, an increase of 28% from 2020, and in British Columbia, Canada, more than 2000 deaths from overdose were reported in 2022 [5–7]. To achieve global HCV elimination by 2030, people who use drugs (PWUD) need to be a priority for engagement and treatment. However, some healthcare professionals (HCPs) treating hepatitis C remain concerned about the potential for drug–drug interactions (DDIs) between opioids, such as fentanyl and DAAs [8]. The Liverpool HEP Drug Interaction Tracker, which relies upon publicly available pharmacokinetic (PK) parameters and prescribing information [9], lists elbasvir/grazoprevir (EBR/GZR) and G/P as having a “potential interaction” when used concomitantly with prescribed fentanyl, oxycodone and hydrocodone, due to weak CYP3A inhibition by the protease inhibitors grazoprevir and glecaprevir, respectively. Product labeling for opioids often includes risk information for concomitant use with CYP3A inhibitors [10–12], though notably these are for strong inhibitors like ketoconazole, itraconazole, and ritonavir, whereas grazoprevir and glecaprevir are weak inhibitors.

[13, 14] Previous PK studies have been performed to explore DDIs between DAAs (including daclatasvir, EBR/GZR, and G/P) and opioids, such as methadone and buprenorphine/naloxone. The results from these studies suggested that PK was not affected when DAAs were used concomitantly with these opioids [15–18]. However, there remains an unmet need to explore any potential impact on safety from DDIs for individuals receiving DAA therapy for HCV infection who are at risk of overdose from opioids such as fentanyl, oxycodone, and hydrocodone. The aim of this analysis was to use publicly available adverse event (AE) reports for opioids and DAAs to explore whether the theoretical risk for potential DDIs has translated into a significant number of events reported in clinical practice, particularly among those who use fentanyl.

## Methods

### Study sample

Data were downloaded from the US Food and Drug Administration (FDA) Adverse Event Reporting System (FAERS) Public Dashboard [19]. AEs with the DAAs G/P, SOF/VEL, ledipasvir/sofosbuvir (LDV/SOF), sofosbuvir/velpatasvir/voxilaprevir (SOF/VEL/VOX), and EBR/GZR listed as the suspect product and an initial received date from July 28, 2017 (the latest global approval date for a pangenotypic DAA regimen) to December 31, 2021, were collected. Data were analyzed in February 2022. Data used in this study are publicly available through the FAERS public dashboard, so ethical approval was not required.

### Measures

To establish a denominator for the total number of AEs with opioids as a suspect product irrespective of HCV treatment, overall AEs for fentanyl, oxycodone, and hydrocodone were counted. The number of AEs and deaths were counted based on concomitant opioid use (either listed as a co-suspect product or as a concomitant medication). Additionally, the number of reports with overdose AEs (reaction terms “overdose,” accidental overdose,” or “intentional overdose”) was counted, irrespective of concomitant opioid use. AEs with opioids of interest (fentanyl, oxycodone, hydrocodone) listed as the suspect product (generic terms only), with an initial received date between 2017 and 2021, were also collected. All search terms used are presented in Additional file 1. In addition, these outcomes were categorized by severity and country/region. Yearly counts of overdoses and fentanyl AEs were assessed in years with complete data, 2018 to 2021.

### Statistical analysis

Descriptive data were reported without additional statistical analysis. Microsoft® Excel (Redmond, WA) was used to perform the analyses and create the figures.

## Results

Between 2017 and 2021, the total number of AEs recorded where fentanyl was the suspect product was 58,001, of which 48,709 (84%) were considered serious and 29,850 (51%) resulted in death. The numbers of AEs where oxycodone or hydrocodone were the suspect product were 290,224. Of these AEs, 274,804 (95%) were serious and 119,013 (41%) resulted in death.

**Fig. 1:**
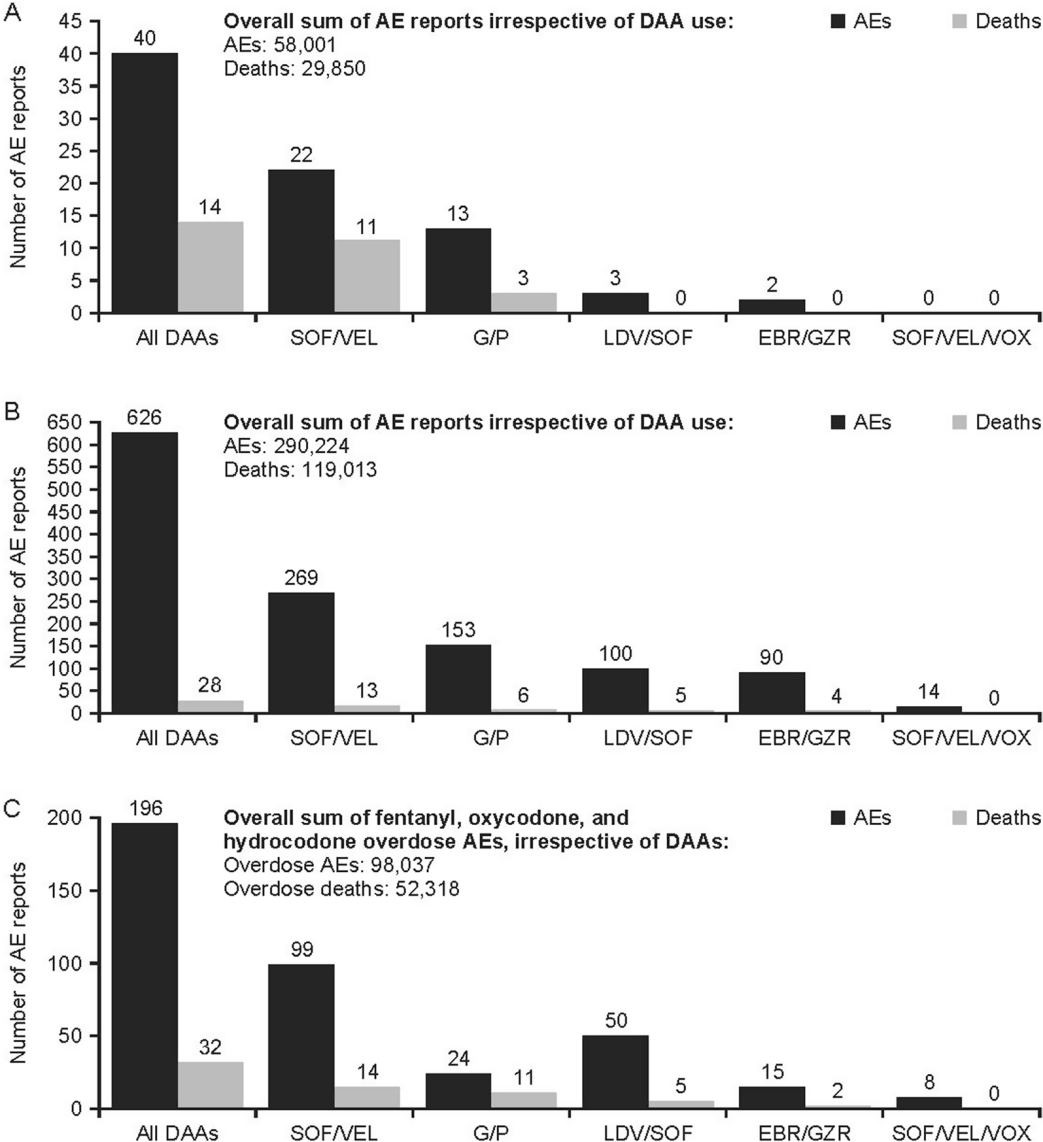
FAERS AE reports. FAERS AE reports for **A** concomitant fentanyl and DAA use **B** concomitant oxycodone/hydrocodone and DAA use **C** overdose AEs and deaths irrespective of concomitant opioid use. AEs, adverse events; DAA, direct-acting antiviral; EBR/GZR, elbasvir/grazoprevir; FAERS, US Food and Drug Administration Adverse Event Reporting System; G/P, glecaprevir/pibrentasvir; LDV/SOF, ledipasvir/sofosbuvir; SOF/VEL, sofosbuvir/velpatasvir; SOF/VEL/VOX, sofosbuvir/velpatasvir/voxilaprevir

During July 28, 2017–December 31, 2021, there were a total of 40 AE reports with concomitant DAA and fentanyl use (SOF/VEL = 22 [55%]), G/P = 13 [33%], LDV/SOF = 3 [8%], EBR/GZR = 2 [5%]); 14 (35%) resulted in death (SOF/VEL = 11 [79%], G/P = 3 [21%]; Fig. 1A). Of the AEs recorded, 35 (88%) were considered serious (SOF/VEL = 20 [57%], G/P = 13 [37%], LDV/SOF = 2 [6%]; Additional file 2). The majority of the AEs recorded were from the USA (25/40 [63%]; Additional file 3: Table S3).

With concomitant DAA and oxycodone/hydrocodone use, 626 AE reports were recorded (SOF/VEL = 269 [43%], G/P = 153 [24%], LDV/SOF = 100 [16%], EBR/GZR = 90 [14%], SOF/VEL/VOX = 14 [2%]); 28 (4.5%) resulted in death (SOF/VEL = 13 [46%], G/P = 6 [21%], LDV/SOF = 5 [18%], EBR/GZR = 4 [14%]; Fig. 1B). In total, 296 (47%) AE reports were recorded as serious, (G/P = 114 [39%], SOF/VEL = 71 [24%], LDV/SOF = 61 [21%], EBR/GZR = 41 [14%], SOF/VEL/VOX = 9 [3%]; Additional file 2) and most AEs were recorded from the USA (569/626 [91%]; Additional file 3).

The total number of overdose reports listing fentanyl, oxycodone, or hydrocodone as suspect products, irrespective of HCV treatment, was 98,037 between 2017 and 2021. This included 10,712 (11%) fentanyl overdose reports (8457 [7%] deaths), 57,629 (59%) oxycodone overdose reports (27,127 [28%] deaths), and 29,696 (30%) hydrocodone overdose reports (16,734 [17%] deaths).

In patients with a DAA listed as the suspect product, 196 overdose reports were recorded (SOF/VEL = 99 [51%], LDV/SOF = 50 [26%], G/P = 24 [12%], EBR/GZR = 15 [8%], SOF/VEL/VOX = 8 [4%]); 32 (16%) resulted in death (SOF/VEL = 14 [44%], G/P = 11 [3%], LDV/SOF = 5 [16%], EBR/GZR = 2 [6%]; Fig. 1C). Of these, 100 (51%) were considered serious and 141 (72%) occurred in the USA (Additional file 4).

**Fig. 2:**
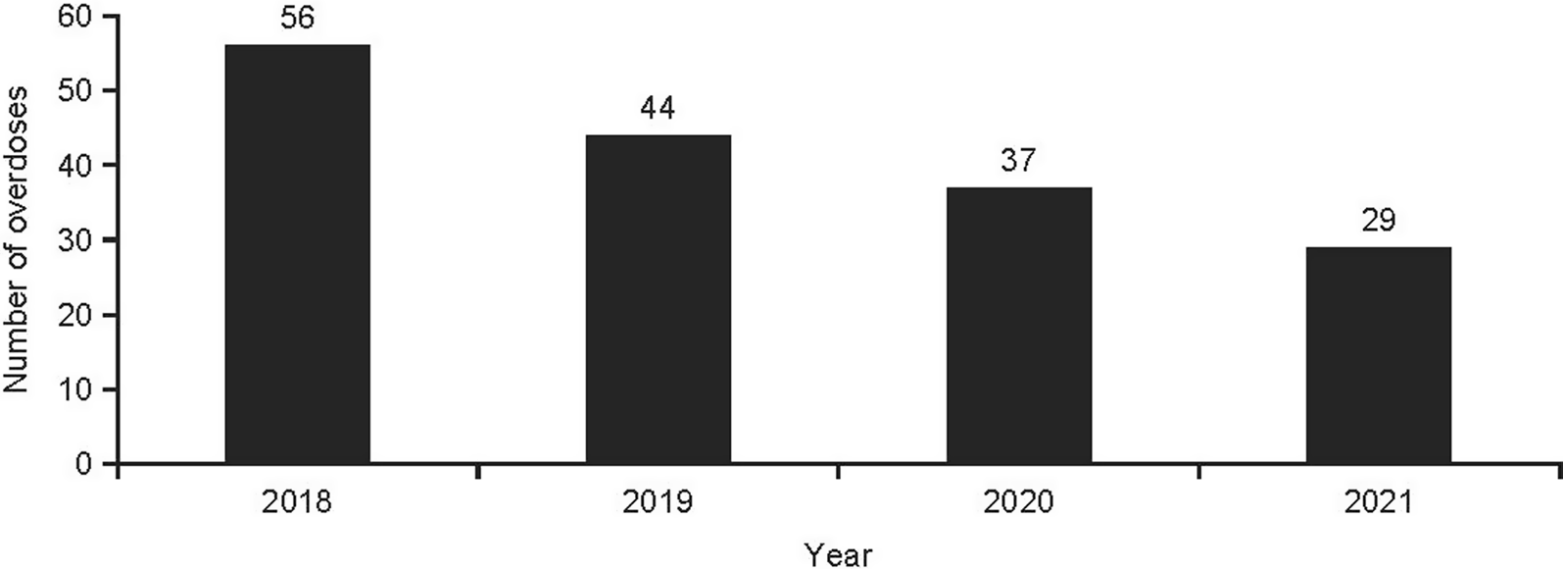
Number of overdose AEs by year in people with a DAA listed as the suspect product. AEs, adverse events; DAA, direct-acting antiviral

The number of overdoses reported declined each year from 2018 (*N* = 56) to 2021 (*N* = 29) (Fig. 2). There were no differences between 2018 (*N* = 9) and 2021 (*N* = 5) in fentanyl AEs.

## Discussion

This analysis found that among ~ 58,000 fentanyl, and ~ 289,000 oxycodone or hydrocodone, AEs reported to FAERS since July 28, 2017, only a very small number (fentanyl: *n* = 40 [0.07%]; oxycodone/hydrocodone: *n* = 626 [0.2%]) have been reported in association with concomitant DAAs, with no link observed between recorded events and any specific DAA regimen, regardless of the theoretical potential for a DDI.

Clinical and PK data related to DDIs between fentanyl, oxycodone, or hydrocodone and modern DAAs are lacking. To our knowledge, there are no clinical or PK studies on coadministration of these opioids with any SOF-based regimen; however, no DDIs are expected for coadministration of these drugs. Concerns related to DDIs between DAAs and opioids are predominantly around the weak CYP34A inhibitors grazoprevir and glecaprevir [15]. A recent physiologically based PK study on the coadministration of G/P with fentanyl suggested that at therapeutic doses of G/P, there is a negligible effect on the PK of IV fentanyl [20]. To date, there are no studies with EBV/GZR and fentanyl, oxycodone, or hydrocodone, but PK studies of other drugs metabolized by CYP34A, methadone, and buprenorphine/naloxone suggest there are no clinically relevant changes in exposure and no dose adjustment is required. [21]

Numerically, most AEs were recorded with SOF/VEL, although no conclusions may be made about this from FAERS data because total concomitant exposures compared to other DAAs are unknown. However, the guideline-recommended first-line pangenotypic DAA regimens SOF/VEL and G/P represent the majority of HCV prescriptions. Over the time period evaluated, SOF/VEL and G/P have accounted for almost equal patient starts in the USA, comprising 46% and 44% of all dispensed DAAs in 2020 [22].

Over the period studied, the number of overdoses with DAAs as the suspect product decreased. A likely reason is that hepatitis C treatment rates in the USA have declined. In 2015, the Centers for Disease Control and Prevention estimated that 164,247 people were treated, and in 2020, this number dropped to 83,740. This reduction in treatment in large part is due to the impact of COVID-19 on healthcare services [23]. Trends in overdose reports and treatment rates over time should be monitored to evaluate any changes in a post-pandemic era once data are available.

## Limitations

Data from this analysis are from one database, which limit international comparisons and overall conclusions. Because of the data source for this study, statistical analysis of the results was not possible. There are several limitations inherent to the FAERS dashboard including the potential for incomplete submissions; inaccurate, untimely, and unverified information; and the inherent underreporting and known duplicate reporting of AEs. The presence of any AE report does not imply causation, and event rates cannot be established due to unknown total exposures.[19] Because estimates for overall numbers of DAA prescriptions are uncertain or unavailable, it is not possible to include information on the proportion of patients prescribed each regimen who had an AE report. Data were restricted to AE reports related to fentanyl, oxycodone, and hydrocodone because PK studies have suggested that no DDIs are expected with opioids such as methadone and buprenorphine/naloxone.

## Conclusions

PWUD, including those who use fentanyl, are a priority patient population for HCV therapy to achieve HCV elimination by 2030. This population has been disproportionately affected by the COVID-19 pandemic and must be prioritized for HCV treatment, regardless of ongoing illicit drug use as recommended by the American Association for the Study of Liver Diseases and Infectious Diseases Society of America (AASLD/IDSA), American Society of Addiction Medicine (ASAM) guidelines, European Association for the Study of the Liver (EASL), and guidelines for Australia [24–27]. As we embrace this call to action, HCPs can be reassured on the low numbers of DAA adverse event reports with opioids.

### Supplementary Information

**Additional file 1**. US Food and Drug Administration Adverse Event Reporting System search terms by substance (DAAs and opioids).

**Additional file 2**. Adverse events by severity for patients with concomitant DAA and opioid use.

**Additional file 3**. Adverse events by country for patients with concomitant DAA and opioid use

**Additional file 4**. Overdose adverse events by severity and country in patients with DAA listed as suspect product.

## Data Availability

AbbVie is committed to responsible data sharing regarding the clinical trials we sponsor. This includes access to anonymized, individual and trial-level data (analysis data sets), as well as other information (e.g., protocols and Clinical Study Reports), as long as the trials are not part of an ongoing or planned regulatory submission. This includes requests for clinical trial data for unlicensed products and indications. This clinical trial data can be requested by any qualified researchers who engage in rigorous, independent scientific research, and will be provided following review and approval of a research proposal and Statistical Analysis Plan (SAP) and execution of a Data Sharing Agreement (DSA). Data requests can be submitted at any time, and the data will be accessible for 12 months, with possible extensions considered. For more information on the process, or to submit a request, visit the following link: https://www.abbvieclinicaltrials.com/hcp/data-sharing/.

## Abbreviations

DAA: Direct-acting antiviral
AE: Adverse event
HCV: Hepatitis C virus
G/P: Glecaprevir/pibrentasvir
SOF/VEL: Sofosbuvir/velpatasvir
WHO: World Health Organization
PWUD: People who use drugs
HCP: Healthcare professional
DDI: Drug–drug interaction
EBR/GZR: Elbasvir/grazoprevir
FDA: Food and Drug Administration
FAERS: Food and Drug Administration Adverse Event Reporting System
LDV/SOF: Ledipasvir/sofosbuvir
SOF/VEL/VOX: Sofosbuvir/velpatasvir/voxilaprevir
AASLD: American Association for the Study of Liver Diseases
IDSA: Infectious Diseases Society of America
ASAM: American Society of Addiction Medicine
