# Supplementary material for: Reported adverse events related to use of hepatitis C virus direct-acting antivirals with opioids: 2017–2021
Source: Harm Reduct J. 2023 Oct 1;20:142. doi: 10.1186/s12954-023-00874-y (PMC10544489; doi:10.1186/s12954-023-00874-y)
Supplement: Supplementary file 1 — Additional file 1. US Food and Drug Administration Adverse Event Reporting System search terms by substance (DAAs and opioids). [file 12954_2023_874_MOESM1_ESM.docx]

**US Food and Drug Administration Adverse Event Reporting System search terms by substance (DAAs and opioids)**

| **Substance** | **Search Terms** |
| --- | --- |
| Fentanyl | Generic names: fentanyl, fentanyl citrate, fentanyl hydrochloride, acetylfentanyl  Brand names: Actiq®, Duragesic®, Sublimaze®, Fentora®, Abstral®, Onsolis™ |
| Hydrocodone | Generic names: Hydrocodone bitartrate, hydrocodone, hydrocodone bitartrate\ibuprofen, acetaminophen\hydrocodone bitartrate, acetaminophen\hydrocodone, hydrocodone,  Brand names: Vicodin®, Norco®, Lortab®, Hysingla®, Zohydro®, Hycodan®, Robidone®, Anexsia®, Co-Gesic®, Hycet®, Hydromet®, Ibudone®, Liquicet®, Lorcet®, Maxidone®, Reprexain™, Rezira®, Tussicaps®, Tussionex®, Vicoprofen®, Vituz®, Xodol®, Zolvit™, Zutripro™, Zydone®, Tylox® |
| Oxycodone | Generic names: Oxycodone hydrochloride, oxycodone, oxycodone terephthalate, acetaminophen\oxycodone hydrochloride, aspirin\oxycodone chloride, acetaminophen\oxycodone hydrochloride\oxycodone terephthalate, naloxone hydrochloride\oxycodone chloride, oxycodone,  Brand names: Oxaydo®, Xtampza™, Oxycontin®, Oxycet®, Percocet®, Percodan®, Roxicet™, Targiniq™, Xartemis®, Targinact®, Oxynorm®, Roxicodone®, Oxyfast™, Oxydose®, Dazidox®, Endone®, Proladone®, Targin®, Troxyca®, Combunox™, Endodan® |
| DAA | Generic names: glecaprevir/pibrentasvir, sofosbuvir/velpatasvir, ledipasvir/sofosbuvir, elbasvir/grazoprevir, and sofosbuvir/velpatasvir/voxilaprevir  Brand names: Mavyret®, Epclusa®, Harvoni®, Zepatier®, Vosevi® |

DAA, direct-acting antiviral; FAERS, US Food and Drug Administration Adverse Event Reporting System
