# Supplementary material for: Reported adverse events related to use of hepatitis C virus direct-acting antivirals with opioids: 2017–2021
Source: Harm Reduct J. 2023 Oct 1;20:142. doi: 10.1186/s12954-023-00874-y (PMC10544489; doi:10.1186/s12954-023-00874-y)
Supplement: Supplementary file 2 — Additional file 2. Adverse events by severity for patients with concomitant DAA and opioid use. [file 12954_2023_874_MOESM2_ESM.docx]

**Adverse events by severity for patients with concomitant DAA and opioid use**

|  |  | **Concomitant fentanyl use** | | |  | **Concomitant hydrocodone/oxycodone use** | | |
| --- | --- | --- | --- | --- | --- | --- | --- | --- |
| **DAA** |  | **All** | **Serious** | **Non-serious** |  | **All** | **Serious** | **Non-serious** |
| **All DAA** |  | 40 | 35 | 5 |  | 626 | 296 | 330 |
| **SOF/VEL** |  | 22 | 20 | 2 |  | 269 | 71 | 198 |
| **G/P** |  | 13 | 13 | 0 |  | 153 | 114 | 39 |
| **LDV/SOF** |  | 3 | 2 | 1 |  | 100 | 61 | 39 |
| **EBR/GZR** |  | 2 | 0 | 2 |  | 90 | 41 | 49 |
| **SOF/VEL/VOX** |  | 0 | 0 | 0 |  | 14 | 9 | 5 |

DAA, direct-acting antiviral; EBR/GZR, elbasvir/grazoprevir; G/P, glecaprevir/pibrentasvir; LDV/SOF, ledipasvir/sofosbuvir; SOF/VEL, sofosbuvir/velpatasvir; SOF/VEL/VOX, sofosbuvir/velpatasvir/voxilaprevir.
