# Supplementary material for: Reported adverse events related to use of hepatitis C virus direct-acting antivirals with opioids: 2017–2021
Source: Harm Reduct J. 2023 Oct 1;20:142. doi: 10.1186/s12954-023-00874-y (PMC10544489; doi:10.1186/s12954-023-00874-y)
Supplement: Supplementary file 3 — Additional file 3. Adverse events by country for patients with concomitant DAA and opioid use [file 12954_2023_874_MOESM3_ESM.docx]

**Adverse events by country for patients with concomitant DAA and opioid use**

|  |  | **Concomitant fentanyl use** | | | | |  | | **Concomitant hydrocodone/oxycodone use** | | | | | |  |
| --- | --- | --- | --- | --- | --- | --- | --- | --- | --- | --- | --- | --- | --- | --- | --- |
| **Country^a^** |  | **All** | **USA** | **CA** | **DE** | **Other** | |  | | **All** | **USA** | **CA** | **DE** | **Other** | |
| **All DAA** |  | 40 | 25 | 10 | 3 | 2 | |  | | 626 | 569 | 8 | 5 | 24 | |
| **SOF/VEL** |  | 22 | 11 | 9 | 0 | 2 | |  | | 269 | 258 | 3 | 0 | 8 | |
| **G/P** |  | 13 | 9 | 1 | 3 | 0 | |  | | 153 | 126 | 1 | 2 | 4 | |
| **LDV/SOF** |  | 3 | 3 | 0 | 0 | 0 | |  | | 100 | 94 | 1 | 1 | 4 | |
| **EBR/GZR** |  | 2 | 2 | 0 | 0 | 0 | |  | | 90 | 78 | 2 | 2 | 8 | |
| **SOF/VEL/VOX** |  | 0 | 0 | 0 | 0 | 0 | |  | | 14 | 13 | 1 | 0 | 0 | |

^a^Origin of the record

CA, Canada; DAA, direct-acting antiviral; DE, Germany; EBR/GZR, elbasvir/grazoprevir; G/P, glecaprevir/pibrentasvir; LDV/SOF, ledipasvir/sofosbuvir; SOF/VEL, sofosbuvir/velpatasvir; SOF/VEL/VOX, sofosbuvir/velpatasvir/voxilaprevir.
