# Supplementary material for: Reported adverse events related to use of hepatitis C virus direct-acting antivirals with opioids: 2017–2021
Source: Harm Reduct J. 2023 Oct 1;20:142. doi: 10.1186/s12954-023-00874-y (PMC10544489; doi:10.1186/s12954-023-00874-y)
Supplement: Supplementary file 4 — Additional file 4. Overdose adverse events by severity and country in patients with DAA listed as suspect product. [file 12954_2023_874_MOESM4_ESM.docx]

**Overdose adverse events by severity and country in patients with DAA listed as suspect product**

|  |  | **Country** | | | | |  | **Severity** | |
| --- | --- | --- | --- | --- | --- | --- | --- | --- | --- |
| **Country**^a^ |  | **All** | **USA** | **CA** | **DE** | **Other** |  | **Serious** | **Non-serious** |
| **All DAA** |  | 196 | 141 | 11 | 2 | 42 |  | 100 | 96 |
| **SOF/VEL** |  | 99 | 70 | 10 | 0 | 19 |  | 41 | 58 |
| **G/P** |  | 24 | 13 | 1 | 1 | 9 |  | 21 | 3 |
| **LDV/SOF** |  | 50 | 39 | 0 | 1 | 10 |  | 30 | 20 |
| **EBR/GZR** |  | 15 | 12 | 0 | 0 | 3 |  | 7 | 8 |
| **SOF/VEL/VOX** |  | 8 | 7 | 0 | 0 | 1 |  | 1 | 7 |

^a^Origin of the record

CA, Canada; DAA, direct-acting antiviral; DE, Germany; EBR/GZR, elbasvir/grazoprevir; G/P, glecaprevir/pibrentasvir; LDV/SOF, ledipasvir/sofosbuvir; SOF/VEL, sofosbuvir/velpatasvir; SOF/VEL/VOX, sofosbuvir/velpatasvir/voxilaprevir.
